# Knowledge, Attitude, and Practice of College Students Toward E-cigarettes: A Study From the Northern Part of Karnataka, India

**DOI:** 10.7759/cureus.68403

**Published:** 2024-09-01

**Authors:** Sreerag Ashok, Santosh Ramdurg, Shivakumar P Chaukimath

**Affiliations:** 1 Psychiatry, Shri BM Patil Medical College Hospital and Research Centre (BLDE), Vijayapura, IND

**Keywords:** knowledge attitude and practice, knowledge, vaping prevention, north karnataka, students and smoking, e-cigarette laws, e-cigarette use in adolescents, cigarette smoking, smoking tobacco, e-cigarette use

## Abstract

Introduction: E-cigarettes, also known as electronic cigarettes or vape pens, are battery-powered devices that deliver nicotine, flavorings, and other chemicals to users in the form of an aerosol. It revolutionized the landscape of nicotine consumption, marketed as a safer alternative to traditional cigarettes. Since then, e-cigarettes have become popular among young adults and adolescents. There has always been a conflict in the risks versus benefits of e-cigarettes over the decade, which has created a gap in knowledge in the population. This study aimed to attain the knowledge, attitude, and practice of students from a semi-urban background to understand the level of education needed among the young population of our country.

Methods: This online survey was conducted among 411 students of Shri BM Patil Medical College Hospital and Research Centre (BLDE) (DU), College, Vijayapura, Karnataka. The survey consisted of 21 MCQ questionnaires to assess students’ knowledge, attitude, and practice toward e-cigarettes. Later, the data was analyzed using SPSS version 28.

Results: The study included a diverse group of 411 participants, with a majority being males (63%) and the rest females (37%). The age distribution highlighted a young demographic, with 93% of participants aged between 18 and 34. The study revealed a high level of awareness about e-cigarettes, with 90% of participants being familiar with them. Friends and social media emerged as the primary sources of information, accounting for 52% and 40%, respectively. While 48% of the participants believed e-cigarettes to be non-addictive, only 19% recognized their addictive potential, with the rest being unsure. A majority, 45%, did not see e-cigarette users as superior to traditional smokers, while 15.5% held the opposite view. While 58% of e-cigarette users found them to be stress-relieving, 33% did not, highlighting a subjective aspect of e-cigarette use.

Conclusion: The study offers critical insights into the knowledge, attitudes, and practices regarding e-cigarettes among medical students in northern Karnataka. While awareness is high, there is a notable gap in accurate understanding of e-cigarettes’ safety and addictive nature. The findings emphasize the need for enhanced education and awareness campaigns to correct misconceptions about e-cigarettes.

## Introduction

E-cigarettes, also known as electronic cigarettes or vape pens, are battery-powered devices that deliver nicotine, flavorings, and other chemicals to users in the form of an aerosol. E-cigarettes were first introduced in the early 2000s as a novel way to deliver nicotine without the harmful combustion products of tobacco. It revolutionized the landscape of nicotine consumption, marketed as a safer alternative to traditional cigarettes. Since then, e-cigarettes have become popular among young adults and adolescents. The e-liquid or e-juice, typically contains nicotine, flavorings, propylene glycol, and vegetable glycerine. Their appeal lies in the variety of flavors and the perception of reduced harm compared to traditional smoking [1,2].

Worldwide, e-cigarette popularity and use among adolescents and children have increased alarmingly. The use of e-cigarettes is common among adolescents in the country, and around 4% of adolescents aged 15-24 years were reportedly aware of e-cigarettes, according to the Global Adult Tobacco Survey (GATS)-2 [3,4]. Currently, more than 460 brands of e-cigarettes with over 7,700 flavors are available for use. E-cigarettes are marketed to youth by promoting flavors through a wide range of media channels, mainly social media [5]. E-cigarette manufacturers are expanding the vaping business by attracting the youth to the new vaping culture by creating a cloud of misperception of harmless and excellent ways of nicotine inhalation [5].

Numerous studies have shown that adolescents are particularly vulnerable to nicotine addiction due to the ongoing development of their brains. Nicotine exposure during adolescence can lead to stronger addiction and long-term cognitive and behavioral changes. The study highlights that the adolescent brain is particularly susceptible to the effects of nicotine because it is still undergoing critical developmental processes, especially in areas like the prefrontal cortex, which is responsible for decision-making, impulse control, and emotional regulation. Nicotine alters the normal development of neural circuits by affecting neurotransmitter systems, particularly acetylcholine receptors, which play a crucial role in brain development. The study found that nicotine exposure during adolescence leads to alterations in the structure and function of these neural circuits. Nicotine exposure during adolescence results in long-lasting changes in brain function that persist into adulthood. These changes can manifest as cognitive deficits, increased susceptibility to addiction, and altered emotional responses [6].

Various studies showcased the e-cigarette as an alternative to tobacco cigarettes, reporting it as an aid in the cessation of smoking, while many other studies report differently [7]. Another study documented a significant increase in the incidence of EC use among young people who have never smoked. Nevertheless, other studies demonstrated its positive effect as a smoking cessation method [8]. There has always been a conflict in the risks versus benefits of e-cigarettes over the decade, which has created a gap of knowledge among people.

This study aimed to attain the knowledge, attitude, and practice of students from a semi-urban background to understand the level of education needed among the young population of our country.

## Materials and methods

Study setting and design

This cross-sectional study was done from March 2024 to May 2024 among students of Shri BM Patil Medical College Hospital and Research Centre (BLDE) (DU), Vijayapura, Karnataka.

Study population

A total of 411 students, including medical undergraduates, postgraduates, allied sciences students, and other professionals, participated in the study.

Inclusion Criteria

Any BLDE college student, older than 18 years, willing to participate in online study was included.

Study tools and data collection

Data were collected through a self-administered online questionnaire designed based on the World Health Organization (WHO) GATS with modifications to suit e-cigarette use and adding some questions to fit our study objectives. It included variables related to the participants’ knowledge, attitude, and practice of ECs and their sociodemographic characteristics. The GATS questionnaire is a standardized tool for monitoring adult tobacco use and assessing tobacco control measures across different countries. The GATS questionnaire is developed with input from international experts. It covers a comprehensive range of topics related to tobacco use, such as prevalence, cessation, second-hand smoke exposure, and public opinions on tobacco control measures. GATS demonstrates good test-retest reliability.

The sample size was calculated using the G-power program, where the expected prevalence of EC smoking was 10% (5% margin of error), and the confidence level was 95% (alpha=0.05). The power of the study was 95%, so the minimal sample size needed for this study was estimated to be 138. We increased the sample size to 411 to compensate for incomplete responses and to better represent our convenient sample.

Google Forms were used to conduct the surveys and collect the participants’ responses, and a convenience sampling strategy was used to invite potential participants via various social media platforms. Twenty-one multiple-choice questions were used; the initial three questions were to understand the sociodemographic details, the following two questions to know about the smoking history, eight questions to assess the knowledge about e-cigarettes, the following five questions about attitude toward e-cigarette use, and the last three questions were about e-cigarette practice.

Statistical analysis

Descriptive statistics in terms of frequencies and percentages, means, and standard deviations were used to describe the criteria of the studied sample. A t-test was performed to analyze knowledge among genders, and the association between smoking practice and attitude toward the ban was assessed using the Chi-square test.

Ethical approval and informed consent

All participants were asked to digitally sign the consent form before starting the survey, and the participants’ details were kept anonymous. The study was approved by the Institutional Ethics Committee of BLDE and granted ethical approval (IEC/025/2023-24) on 10th February 2024.

## Results

Out of 411 participants, 257 (63%) were males, and 154 (37%) were females, of which 385 (93%) were in the age group of 18-34 years, and the rest were above 34 years. In the study, 97 (23%) were attaining medical postgraduation degrees, 244 (59%) were medical undergraduate students, and the remaining 70 (17%) were paramedical and professional degree subject students. In this study, we found out that 235 (57%) of them were non-smokers, 176 (43%) of them were smokers, 143 (81%) of them were tobacco smokers, 22 (13%) of them were e-cigarette smokers, and 11 (6%) of them smoke both. Among the study population, 79 of the subjects have used an e-cigarette at least once in the past. Among them, 64 were male and 15 were female. Most have used e-cigarettes within the last year (Table 1).

**Table 1:** Frequency distribution of socio-demographic details and smoking history of participants

Variables	Frequency (n=411)	Percentage (%)
Male	257	63
Female	154	37
Age		
18-34 years	385	93
34-40 years	26	7
Smoking history		
Nonsmokers	235	57
Current smokers (including tobacco and e-cigarettes) (n=411)	176	43
Tobacco smokers (n=176)	143	81
E-cigarette users (n=176)	22	13
Combined (Tobacco and e-cigarette) (n=176)	11	6
Used e-cigarette once in a life-time	79	19

The study revealed that 362 (90%) of them are aware of e-cigarettes, 191 (52%) came to know about it from friends, 144 (40%) from social media, and the rest from other sources. In the study, 139 (33%) believe e-cigarettes to be safer than tobacco cigarettes; the rest of the participants either do not feel it to be safer or do not know. Most of the participants came to know about it within the last five years. 196 (48%) of the total study participants mentioned it to be non-addictive, whereas only 79 (19%) felt it was addictive. In the study, 158 (38%) know it is not legal in India, where 126 (30%) believe it to be legal, and the rest do not know the legality (Table 2).

**Table 2:** Frequency distribution of participants' knowledge of e-cigarette

Knowledge about e-cigarettes			
	Questions	Frequency	Percentage (%)
Knows about e-cigarettes (n=411)	yes	362	88
	No	49	12
Is e-cigarette harmful (n=411)?	Yes	108	27
	No	38	9
	Don’t know	265	64
Is e-cigarette safer than tobacco cigarette (n=411)?	Yes	139	39
	No	132	32
	Don’t know	140	34
Is e-cigarette addictive (n=411)?	Yes	79	19
	No	196	48
	May be	136	33
Is e-cigarette legal in India (n=411)?	Yes	126	31
	No	158	38
	Don’t know	127	31
Known about e-cigarettes from (n=362)?	Friends	191	46
	Social media	144	35
	Advertisement	17	4
	Family members	10	2
Known about e-cigarettes from (n=362)?	Less than 5 years	335	93
	More than 5 years	27	7

As per the study, 211 (51%) of participants had a trying curiosity for the first time, and 170 (41%) participants think e-cigarette helps in quitting tobacco cigarettes. In comparison, 125 (30%) feel it does not have any tobacco cessation benefit. 186 (45%) participants feel e-cigarette users are not superior to conventional smokers, while 64 (15.5%) feel e-cigarettes are superior to tobacco cigarettes. 247 (60%) participants feel it should be banned in India (Table 3).

**Table 3:** Frequency distribution of participants' attitude toward e-cigarette

Attitude toward e-cigarette		
Do you wish to try e-cigarettes (n=411)?	Yes	211
	No	200
Does it help to quit tobacco cigarettes (n=411)?	Yes	170
	No	125
	May be	116
Superiority over smokers (n=411)	Agree	64
	Disagree	186
	Neutral	161
Need to be banned (n=411)	Yes	247
	No	164

Among the 79 participants who tried e-cigarettes, 49 (62%) liked it. Flavor or aroma was the main reason for them to enjoy it, followed by the enjoyment they received while vaping. The study found that 11 (33%) of participants do not feel it is a stress reliever, unlike tobacco cigarettes, while 19 (58%) of participants who use e-cigarette feels it is a stress buster. In the study, we found that 22 (67%) feel it is more pleasurable than a tobacco cigarette to use, while 11 (33%) feel it is not as pleasurable as a conventional tobacco cigarette (Table 4).

**Table 4:** Frequency distribution of participants’ practice toward e-cigarettes

Practice toward e-cigarette			
	Response	Frequency	Percentage
First experience (n=79)	Liked	49	62
	Didn’t like	30	38
Do you prefer to use it over tobacco cigarettes (n=79)?	Yes	49	62
	No	19	24
	Maybe	11	14
Does it help to relieve stress (n=33)?	Yes	19	58
	No	11	33
	Not always	3	9
Pleasurable than tobacco cigarettes (n=33)	Yes	22	67
	No	11	33
Why it has been used (n=79)?	Flavor	34	43
	Vaping process	19	24
	Friends use it	12	15
	Can be used at any place despite tobacco ban	8	10
	To quit smoking	6	8

In our study, we performed a t-test to look for possible significance between knowledge about e-cigarettes and gender; it was found to have no significance between the two (p: 0.841). On checking the significance between smoking history and the attitude of participants toward banning e-cigarettes, we found no statistical significance (p: 0.139) (Table 5).

**Table 5:** Significant factors associated with knowledge, attitude, and practice among study participants about genders and attitudes toward banning and smoking history

Test type	Variables	Test statistic	p-value
t-test	Knowledge by gender	0.2008	0.841
Chi-square test	Smoking history versus attitude to ban	2.191	0.139

## Discussion

E-cigarettes, also known as electronic cigarettes or vape pens, have gained popularity as an alternative to traditional smoking. They are often marketed as a less harmful way to consume nicotine, but their health impacts and safety are still subjects of ongoing research and debate. This study will help explore students’ knowledge, attitude, and practice toward e-cigarettes, mainly from students of the northern part of Karnataka. In the last five years, e-cigarettes have become increasingly popular among young people, mainly due to peer consumption and social media influence. E-cigarette content on social media that promotes e-cigarette flavors and e-cigarettes as cleaner and a socially more acceptable alternative to cigarettes may work to escalate e-cigarette use among experimenters.

Four hundred eleven students participated in the survey, where 257 (63%) were males and 154 (37%) were females; the majority of whom were 18-34 years old. 97 (23%) were attaining medical postgraduation degrees, 244 (59%) were medical undergraduate students, and the remaining 70 (17%) were paramedical and professional degree subject students. The majority of participants, 235 (57%), were nonsmokers. Among the study participants, 79 (19%) of the subjects have used an e-cigarette at least once in the past.

In this study, we found that 362 (88%) of participants are familiar with e-cigarettes, and most were introduced to them by peer and social media. Most of them learned about it within the last five years. A study in 2022 on 24 school students from Mumbai identified that most participants had heard of e-cigarettes, primarily through peers, social media, and advertisements [9]. On assessing the knowledge about the health implications of e-cigarettes, it was found that most of them had limited and often inaccurate information, which was consistent with our study. In the survey, we can see that there is a widespread belief that e-cigarettes are less harmful than traditional cigarettes or most of the students do not know the damaging effects of e-cigarettes, unlike tobacco cigarettes. This all leads to the trying curiosity in the young population [9]. Curiosity and the desire to experiment were primary reasons for initial use; peer pressure and the influence of social media also played significant roles in it. Friends and peer groups were the most critical influence on e-cigarette use; adolescents often started using e-cigarettes to fit in or due to direct encouragement from peers, and his study also discussed the high-risk family influence in starting using e-cigarettes if family members are e-cigarette users [9]. However, our study revealed that the majority of them do not believe that e-cigarettes are superior to tobacco cigarettes nor is it something that they would consider to be fit for the group; this discrepancy might be due to the low socio-economic factor and the less modernized society of study sites. 

In this study, participants tend to have limited knowledge about the harmfulness of e-cigarettes; 74% of participants feel it is safe or do not know the side effects they may cause. On looking at the knowledge of students about safety profiles, 33% feel it is safer than tobacco cigarettes, and 34% do not know whether it is safe or harmful. The majority of participants feel that it does not cause any addiction to using e-cigarettes. The marketing strategies played an excellent role in making the young population create a perception of e-cigarettes as an addiction-free smoking method. Global studies show that a high percentage of participants are aware of e-cigarettes. Still, detailed knowledge varied with knowledge about the potential health risks and benefits of e-cigarettes was mixed, with some misconceptions prevalent. Another study in 2020 also concluded similar results; a significant portion of participants believed e-cigarettes are less harmful than traditional cigarettes, but there were concerns about their long-term health effects; e-cigarettes were generally acceptable, particularly for smoking cessation [10].

In all the studies, including ours, we found that younger age groups and males were more likely to have tried e-cigarettes and had a more favorable attitude toward them. When we look into the knowledge regarding the addictive propensity of e-cigarettes, the majority of the subjects (47%) think it is not addictive, and another 33% do not know whether it is addictive or not. A study in 2018 says e-cigarettes can deliver nicotine levels comparable to traditional cigarettes [11]. The efficiency of nicotine delivery in some e-cigarettes suggests a high potential for addiction. Another study in 2016 examined the pharmacokinetics of nicotine from e-cigarettes and found that nicotine absorption can be rapid and efficient, similar to traditional cigarettes. This fast delivery contributes to the addictive potential of e-cigarettes [12]. A study in 2015 revealed that college students often perceive e-cigarettes as less harmful and more socially acceptable, which contributes to frequent use and addiction. The ease of use and concealability of e-cigarettes were also factors in their addictive potential. All these studies provide us with information that e-cigarettes are addictive and can be a gateway to the use of tobacco cigarettes [13]. In our study, we see a significant lack of knowledge among students regarding the addictive propensity of e-cigarettes. Hence, most of the young population has a perception that it is less harmful and non-addictive compared to tobacco cigarettes.

E-cigarettes were banned in India on September 18, 2019. The Prohibition of Electronic Cigarettes (Production, Manufacture, Import, Export, Transport, Sale, Distribution, Storage and Advertisement) Bill, 2019 was introduced and subsequently passed to formalize the ban [14]. However, our study gives us insight into the fact that only 30% of the study population is aware of it; the rest of them need to be made aware of the initiatives the government has put forth to secure the health of our population. This is not restricted to this study population; first, the reason for this can be the lack of effective communication: The government’s communication regarding the ban has not been widespread or persistent enough to reach all population segments. While the initial announcement received some media coverage, continuous public awareness campaigns have been lacking. Second, there is inconsistent enforcement: The enforcement of the ban has been inconsistent across different regions. In some areas, authorities have not actively pursued enforcement, leading to the continued availability and sale of e-cigarettes. This inconsistency undermines the effectiveness of the ban and contributes to public unawareness. Third, online sales: E-cigarettes are still accessible through online platforms, including e-commerce websites and social media, where enforcement is more challenging. This ease of access undermines the ban and leads to continued use and unawareness among consumers. Last but not least is the media influence: The portrayal of e-cigarettes in media and advertisements before the ban contributed to their popularity. Despite the ban, lingering positive perceptions continue to influence public attitudes, reducing the perceived importance of adhering to the ban [14].

When we look into the practice of students on e-cigarettes, we observed that 62% of students who tried an e-cigarette liked it on first use, and those who liked prefer it over tobacco cigarette; the main reasons for wanting it on first use can be attributed to the flavor and aroma it poses, which is an excellent factor for even nonsmokers to try it. Along with this, many of the e-cigarette smokers or attempters are influenced by the vaping process and stunt shows they can make with it; this was influenced heavily by the social media and advertisements where it was showcased as a nonaddictive, cool, and better alternative to smoking a tobacco cigarette. When looking into the satisfaction of users, we see 66% of students are satisfied, and they feel it is more pleasurable than tobacco cigarettes; high levels of satisfaction of users are due to the variety of flavors, the perceived safety compared to traditional cigarettes, and the ability to control nicotine intake.

On seeing the stress relieving factor, 55% of those who are using e-cigarettes feel that it will relieve stress, while 33% feel it is not an excellent option to reduce stress like tobacco cigarettes. At the same time, 12% feel it does not help reduce stress. A study in 2019 found that e-cigarettes were found to be almost twice as effective as nicotine-replacement therapy in helping participants quit smoking [15]. A study in 2011 reported positive experiences with e-cigarettes, often highlighting their satisfaction with nicotine delivery, the similarity to smoking traditional cigarettes, and the variety of flavors available [16]. Thus, we can conclude that e-cigarette helps relieve stress, but the level of stress reduction may not be the same as that of tobacco cigarettes due to the low nicotine content they possess. 

## Conclusions

E-cigarettes have become popular as an alternative to traditional smoking. Most were introduced to e-cigarettes by peers and social media within the last five years. Even though their safety and health impacts are still under research and debate, the advertisement and marketing portray it as a healthy and safer alternative to tobacco cigarettes. The study provides valuable insights into the knowledge, attitudes, and practices regarding e-cigarettes among the medical population in northern Karnataka. Despite high awareness, there is a significant gap in accurate knowledge about the safety and addictive nature of e-cigarettes. The mixed opinions on their efficacy as a smoking cessation tool and their perceived benefits as stress relievers underscore the complexity of the issue. The findings highlight the need for more comprehensive education and awareness campaigns to address misconceptions about e-cigarettes, as this study portrays the lack of knowledge in the medical population, who are the cornerstone of spreading knowledge to patients and the community. The study emphasizes the addition of side effects of e-cigarettes and their harmfulness in the medical curriculum of our country, which in turn creates concrete knowledge among medical students and helps spread awareness in the community over time. Moreover, consistent enforcement of regulations and effective communication about the legal status of e-cigarettes are crucial to ensuring informed decision-making among young adults. As the debate on e-cigarettes continues, studies like this play a pivotal role in shaping public health policies and interventions to safeguard the population’s well-being. It is hard to spread among the general population when the medical population is unaware of its risks and side effects. In turn, the myths regarding this could never be spread among the current and future generations.

## Appendices

The questionnaire was used to collect the socio-demographic and smoking history to assess the knowledge, attitude, and practice of students about e-cigarettes (designed based on the World Health Organization (WHO) Global Adult Tobacco Survey (GATS) with modifications to suit e-cigarette use) [17].

Choose one response for each question 

1. Age

a) 18-34 years

b) 34-40 years

2. Sex

a) Male

b) Female

3. Course you are studying

a) Medical undergraduate

b) Medical postgraduate

c) Allied health science

d) Nursing

e) Other professional degree

4. Have you smoked e-cigarettes in the past?

a) Yes

b) No

5. Do you currently smoke (If yes, what)?

a) Don’t smoke

b) Smokes tobacco cigarette

c) Uses e-cigarette only  

d) Tobacco cigarette + e-cigarette

6. Before today, have you ever heard about e-cigarettes?

a) Yes

b) No

7. Where did you learn about e-cigarettes?

a) Social media  

b) Online advertising  

c) Family members

d) Friends

8. For how many years are you familiar with e-cigarettes?

a) Less than five years

b) More than five years

9. Have you been curious about using an e-cigarette since seeing it first?

a) Yes

b) No

10. Why do you think people use e-cigarettes?

a) To quit smoking tobacco cigarettes

b) Because they enjoy vaping 

c) You can use it at times when or in places where tobacco smoking is not allowed

d) It is less harmful than smoking tobacco

e) It comes in flavors I like

f) A friend or family member uses them

11. Do you think E-cigarettes give more pleasure than tobacco cigarettes?

a) Yes

b) No

12. Are e-cigarette smoking harmful to health?  

a) Yes

b) No

c) Don’t Know

13. Are e-cigarettes less harmful than tobacco cigarettes?  

a) Yes

b) No

c) Doesn’t Know

14. Is e-cigarettes addictive?

a) Yes

b) No

c) Doesn’t Know

15. Does e-cigarettes pose a lower risk for cancer than traditional cigarettes?  

a) Yes

b) No

c) Doesn’t Know

16. Do you believe e-cigarettes are a helpful aid for smoking cessation?  

a) Yes

b) No

c) Doesn’t Know

17. If I use e-cigarettes, will it gain superiority among my smoking friends?

a) Agree

b) Disagree

c) Neutral

18. Do e-cigarettes give me more pleasure than tobacco cigarettes?

a) Yes

b) No

c) Maybe

19. Do e-cigarettes relieve stress after using them?  

a) Yes

b) No

c) Sometimes, maybe

20. Are e-cigarettes legal in India?  

a) Yes

b) No

c) Doesn’t Know

21. Should e-cigarettes be banned?  

a) Yes

b) No
